# Transcriptomics identifies STAT3 as a key regulator of hippocampal gene expression and anhedonia during withdrawal from chronic alcohol exposure

**DOI:** 10.1038/s41398-021-01421-8

**Published:** 2021-05-20

**Authors:** Wei-Yang Chen, Hu Chen, Kana Hamada, Eleonora Gatta, Ying Chen, Huaibo Zhang, Jenny Drnevich, Harish R. Krishnan, Mark Maienschein-Cline, Dennis R. Grayson, Subhash C. Pandey, Amy W. Lasek

**Affiliations:** 1 Center for Alcohol Research in Epigenetics, Department of Psychiatry, University of Illinois at Chicago, Chicago, IL 60612 USA; 2 Roy J. Carver Biotechnology Center, University of Illinois at Urbana-Champaign, Urbana, IL 61801 USA; 3 Research Informatics Core, University of Illinois at Chicago, Chicago, IL 60612 USA; 4 Jesse Brown VA Medical Center, Chicago, IL 60612 USA

**Keywords:** Molecular neuroscience, Addiction

## Abstract

Alcohol use disorder (AUD) is highly comorbid with depression. Withdrawal from chronic alcohol drinking results in depression and understanding brain molecular mechanisms that drive withdrawal-related depression is important for finding new drug targets to treat these comorbid conditions. Here, we performed RNA sequencing of the rat hippocampus during withdrawal from chronic alcohol drinking to discover key signaling pathways involved in alcohol withdrawal-related depressive-like behavior. Data were analyzed by weighted gene co-expression network analysis to identify several modules of co-expressed genes that could have a common underlying regulatory mechanism. One of the hub, or highly interconnected, genes in module 1 that increased during alcohol withdrawal was the transcription factor, signal transducer and activator of transcription 3 (*Stat3*), a known regulator of immune gene expression. Total and phosphorylated (p)STAT3 protein levels were also increased in the hippocampus during withdrawal after chronic alcohol exposure. Further, pSTAT3 binding was enriched at the module 1 genes *Gfap, Tnfrsf1a*, and *Socs3* during alcohol withdrawal. Notably, pSTAT3 and its target genes were elevated in the postmortem hippocampus of human subjects with AUD when compared with control subjects. To determine the behavioral relevance of STAT3 activation during alcohol withdrawal, we treated rats with the STAT3 inhibitor stattic and tested for sucrose preference as a measure of anhedonia. STAT3 inhibition alleviated alcohol withdrawal-induced anhedonia. These results demonstrate activation of STAT3 signaling in the hippocampus during alcohol withdrawal in rats and in human AUD subjects, and suggest that STAT3 could be a therapeutic target for reducing comorbid AUD and depression.

## Introduction

Withdrawal from chronic alcohol drinking elicits negative emotional states such as anxiety, anhedonia, and depression^1,2^. These psychological states are detrimental to achieving abstinence from alcohol use and are associated with increased risk for relapse to alcohol drinking^3,4^. The hippocampus is an alcohol-sensitive brain region involved in mood regulation and cognition^5–8^. Brain imaging of human subjects with alcohol use disorder (AUD) has found reductions in hippocampal volume and altered hippocampal connectivity^7^, which likely contribute to cognitive and emotional problems observed in those with AUD. In rodent models of AUD, chronic alcohol exposure and withdrawal alters the hippocampus at the cellular and molecular level^9–15^. Chronic alcohol drinking increases the expression of inflammatory mediators, such as cytokines and chemokines, increases oxidative stress, and induces excitotoxicity and reactive gliosis^16,17^. This constellation of changes is associated with altered synaptic function, reduced neurogenesis, and ultimately, neurodegeneration^16^, as well as increased depressive-like behaviors and learning and memory deficits during alcohol withdrawal^10,18,19^.

Activation of the innate immune system during withdrawal from chronic alcohol use is particularly interesting because an overactive immune response has also been observed in human subjects with major depressive disorder^20,21^, and in rodent models of stress-induced depression^21^, suggesting that a brain pro-inflammatory response might be a common mechanism linking AUD and depression during alcohol withdrawal. Cytokines and chemokines bind to cell surface receptors, resulting in the activation and nuclear translocation of transcription factors such as the nuclear factor of kappa light polypeptide gene enhancer in B cells (NF-κB) complex and signal transducer and activator of transcription 3 (STAT3)^22,23^. These transcription factors bind to specific DNA sequences to increase the expression of genes involved in the immune response, such as chemokines, cytokines, and their receptors, thus amplifying the immune response. Activation of the NF-κB complex has been observed in the hippocampus of human subjects with AUD and in rodent models of AUD^24,25^. Interestingly, STAT3 plays a role in depressive-like behaviors in mice^26,27^, and polymorphisms in the human *STAT3* gene are associated with the response to antidepressants^28^. Acute and chronic alcohol exposure activates STAT3 in the mouse hippocampus^29,30^. However, it is unknown whether STAT3 in the hippocampus regulates expression of immune response genes and development of depression-like behaviors during ethanol withdrawal after chronic exposure.

We therefore used an unbiased transcriptomic approach to identify gene expression changes in the rat hippocampus during chronic ethanol exposure and withdrawal, with the aim of identifying genes and signaling pathways that could contribute to alcohol withdrawal-induced depressive-like behavior. We identified STAT3 as a “hub” gene that is increased in the hippocampus during alcohol withdrawal. STAT3 was activated in hippocampal astrocytes during alcohol withdrawal and was more highly associated with the promoter of glial fibrillary acidic protein (*Gfap*), which is a hallmark of activated astrocytes, and the promoters of several immune-related genes whose expression also increased during alcohol withdrawal. We used a pharmacological inhibitor of STAT3 to demonstrate that activation of STAT3 during alcohol withdrawal is behaviorally relevant to anhedonia, a key symptom of depression^31^. Finally, we examined the activation of STAT3 and gene expression in the postmortem hippocampus of human AUD subjects.

## Materials and methods

### Animals

Adult male Sprague Dawley rats (75–85 days old) were purchased from Harlan (Indianapolis, IN, USA) and individually housed in a temperature- and humidity-controlled room with a 12-h light/dark cycle (lights on a 6 am), with food and water provided ad libitum prior to beginning the chronic ethanol treatment protocol. Animal care was conducted according to the National Institutes of Health *Guide for the Care and Use of Laboratory Animals* and all experimental procedures were approved by the University of Illinois at Chicago Animal Care Committee.

### Chronic ethanol treatment

When rats reached ~250–300 g body weight, they were fed with the nutritionally complete Lieber-DeCarli control or ethanol liquid diet (Bio-Serv; Frenchtown, NJ, USA) as their only source of food and fluid as described by us previously^32,33^. Rats were randomly assigned to treatment groups. Rats were fed 80 ml/day control diet for 3 days and then were randomly assigned to different treatment groups. The control group continued with the control diet for 16 days, while the ethanol groups were gradually introduced to ethanol over 7 days (1.8–8.1%). Rats were then maintained on a 9% ethanol diet for 15 days. Ethanol-withdrawn rats were switched to control liquid diet for 24 h after removal of the ethanol liquid diet whereas ethanol diet-fed (0 h withdrawal) continued on an ethanol diet for one additional day. Rats were pair fed and their liquid diet intake and body weights were closely monitored. We have previously reported blood alcohol levels (BALs) in the range of 172–198 mg% for ethanol-diet fed (no withdrawal) rats, whereas the BALs after 24 h of ethanol withdrawal were undetectable^32,33^. Investigators were not blinded to treatment groups.

### Tissue collection

Rats were anesthetized with isoflurane and rapidly decapitated. Brains were removed and sectioned into a 1-mm-thick section using a rat brain matrix (Zivic Instruments, Pittsburgh, PA, USA). The dorsal hippocampus (located 2–4 mm posterior to bregma) was dissected, rapidly frozen on dry ice and stored at −80 ^o^C.

### RNA-Seq and bioinformatics

RNA-Seq was performed on dorsal hippocampi from 6 rats per group (control, ethanol, and withdrawal). Total RNA was isolated using the miRNeasy Mini Kit (Qiagen) and RNA integrity numbers were determined using a TapeStation instrument (Agilent, Santa Clara, CA, USA). cDNA libraries were prepared using the TruSeq RNA Library Preparation Kit (Illumina, San Diego, CA, USA) and were paired-end sequenced on the HiSeq 4000 System (Illumina) Approximately 100,000,000 raw reads were obtained per sample. The libraries were prepared and sequenced in the DNA Services laboratory of the Roy J. Carver Biotechnology Center at the University of Illinois at Urbana-Champaign. Sequences were aligned to the NCBI Rnor 6.0 genome (annotation 106) using STAR (v. 2.5.2a). A total of 18,118 genes had edgeR’s^34^ TMM-normalized values >0.5 and were kept for differential expression (DE) analysis. After adjusting for surrogate variables^35^, we used limma-voom^36^ to calculate a one-way ANOVA across all three treatments. Weighted gene co-expression network analysis (WGCNA) was performed in order to group genes into modules showing similar expression patterns across samples. Principle components analysis of genes in modules was used to calculate the “eigengene” value. Clusters or modules with similar expression patterns were merged, resulting in a total of 53 modules. STRING^37^ and Enrichr^38^ were used for pathway analysis. RNA-Seq data are available in Gene Expression Omnibus (GEO) database, accession number GSE171051.

### Quantitative real-time PCR (qPCR)

qPCR was performed on RNA from dorsal hippocampi from 10 rats per group (control, ethanol, and withdrawal; six were samples from the RNA-Seq experiment plus an additional four samples). Total RNA was isolated from frozen tissue using TRIzol (Thermo Fisher Scientific), further purified using the miRNeasy Mini kit (Qiagen) and converted to cDNA using iScript Reverse Transcription Supermix (Bio-Rad, Hercules, CA, USA). For postmortem hippocampus tissue, qPCR was performed in 13 controls, 13 AUD subjects with no blood alcohol levels at the time of death, and 7 AUD subjects with positive blood alcohol level (demographic details are in Table 1). Total RNA was isolated from frozen tissue using the miRNeasy Mini kit (Qiagen) then converted to cDNA using M-MLV Reverse Transcriptase (Invitrogen). SYBR Green Supermix (Bio-Rad) was used for qPCR with the primers listed in Supplemental Methods.

**Table 1:** Demographic characteristics of control and alcohol use disorder (AUD) subjects.

	Samples used for pSTAT3 IHC		Samples used for qPCR		
Subjects	Control	AUD	Control	AUD	AUD-BAL
*n*	14	14	13	13	7
Age	61.6 ± 2.2	61.1 ± 2.4	57 ± 2	60 ± 1.8	49 ± 2.8^#^
Sex (*n*)	F (4), M (10)	F (4), M (10)	F (2), M (11)	F (4), M (9)	F (1), M (6)
PMI (h)	34.4 ± 3.7	36.7 ± 3.8	35 ± 4.2	39 ± 5	40 ± 4.4
pH	6.6 ± 0.08	6.6 ± 0.06	6.6 ± 0.08	6.6 ± 0.08	6.7 ± 0.16
BMI	31.3 ± 1.7	25.8 ± 1.4*	31 ± 1.5	25 ± 1.9	29 ± 2.0
Total drinking years	36.9 ± 3.3	35.9 ± 2.7	35 ± 3.1	38 ± 1.8	28 ± 3.2
Ethanol daily use (g)	17.5 ± 5.6	159.8 ± 32.6***	14 ± 4.9	179 ± 35**	258 ± 73***
Drinks per week	10.9 ± 4.7	78.7 ± 18.2**	8.5 ± 3.6	87 ± 19*	147 ± 53**
Cigarette pack years	16.9 ± 6.6	40.0 ± 7.3*	25 ± 9.7	39 ± 5.1	30 ± 4.7
Cause of death (*n*)	Cardiac (10), cancer (1), cardiovascular (1), respiratory (1), vascular (1)	Cardiac (8), hepatic (1), infection (2), respiratory (2), toxicity (1)	Cardiac (10), vascular (1), cardiovascular (1), toxicity (1)	Cardiac (6), respiratory (3), hepatic (1), toxicity (1), infection (1), stroke (1)	Cardiac (3), toxicity (3), unknown (1)

Data are shown as mean ± SEM.

*BMI* body mass index, *F* female, *M* male, *PMI* postmortem interval (hours).

*p < 0.05, **p < 0.01, ***p < 0.001 by unpaired two-tailed Student’s *t*-test when compared to control. ^#^*p* < 0.01 when compared to AUD.

### Immunohistochemistry (IHC)

Rats (3 per group) were euthanized using a commercial euthanasia solution containing pentobarbital and transcardially perfused with 4% paraformaldehyde. Brains were processed for IHC with antibodies to phosphorylated (p)STAT3 (phosphorylated at tyrosine 705, Cell Signaling Technology, RRID: AB_2198588) and GFAP (Thermo Fisher Scientific, RRID: AB_2532994) as detailed in Supplemental Methods. Human brain slices (10 male and 4 female from each group) were deparaffinized and heated for antigen retrieval. The same pSTAT3 antibody and method used for rat sections were performed on human slices.

### Western blot

Western blots were performed on hippocampal lysates from 6 rats per group using standard procedures with primary antibodies to STAT3 and pSTAT3 (Cell Signaling Technology, RRID: AB_331757 and RRID: AB_2491009), and β-actin (Sigma-Aldrich RRID: AB_476744) as detailed in Supplemental Methods. Blots were imaged on the Odyssey Fc Dual-Mode Imaging system (LI-COR, Lincoln, NE, USA).

### Chromatin immunoprecipitation (ChIP)

ChIP was performed on dorsal hippocampi from 14 rats per group. Frozen tissue was homogenized in phosphate-buffered saline (PBS) and fixed in 1% methanol-free formaldehyde for 5 min at 37 ^o^C. Homogenate was lysed with SDS buffer (1% SDS, 50 mM Tris-HCl, pH 8, 10 mM EDTA) for 10 min at 4 ^o^C and sonicated (Covaris, Woburn, MA, USA) to shear chromatin to 200 bp fragments. Chromatin (100 μg) was diluted in ChIP dilution buffer (0.01% SDS, 1.1% Triton X-100, 1.2 mM EDTA, 16.7 mM Tris-HCl, pH 8, 167 mM NaCl) and an aliquot was removed as input. Samples were pre-cleared with protein A/G PLUS-agarose beads (Santa Cruz Biotechnology, Dallas, TX, USA) prior to immunoprecipitation (IP). IP was performed overnight at 4 ^o^C with an antibody to pSTAT3 (5 μl, #9145; Cell Signaling Technology; RRID: AB_2491009) and protein A/G PLUS-agarose. IPs were washed with ChIP dilution buffer and chromatin isolated using Chelex 100 resin (Bio-Rad). qPCR was performed as described above with the primers indicated in Supplementary methods. Relative enrichment of pSTAT3 binding was calculated by normalizing to input using 2^−ΔΔCT^ method.

### Sucrose preference test

Sucrose preference testing was performed on 14–16 rats per group (control-vehicle *n* = 16, withdrawal-vehicle *n* = 14, control-stattic *n* = 16, and withdrawal-stattic *n* = 16). Bottles containing control liquid diet were removed from the home cages at 24 h of withdrawal (of both ethanol-withdrawn and control rats) and replaced with two standard water bottles containing either a 0.5% sucrose (w/v) solution or water for 1 h. Stattic (Selleck Chemicals, Houston, TX, USA) was administered intraperitoneally at a dose of 50 mg/kg in vehicle (2% DMSO, 30% PEG300), 2 h prior to testing sucrose preference. Dorsal hippocampi were dissected after the completion of the sucrose preference test and subjected to either western blots or ChIP with pSTAT3 antibody to demonstrate stattic efficacy.

### Human subjects

Postmortem hippocampal sections and frozen hippocampus from subjects diagnosed with AUD and control subjects (*n* = 10 males and 4 females per group for IHC; *n* = 13 control, 13 AUD, and 7 AUD with positive blood alcohol levels for qPCR) were obtained from the New South Wales Brain Tissue Resource Centre (University of Sydney, Australia). Demographic characteristics of subjects are shown in Table 1. Individuals were diagnosed with AUD according to DSM-IV criteria. Control cases were matched with AUD for sex, age, race, and postmortem interval. IHC was used for pSTAT3 staining in sections as described above. One AUD subject was removed from the pSTAT3 analysis because it was identified as a statistical outlier using the ROUT method (*Q* = 1%). RNA was isolated from frozen tissue and analyzed by qPCR as described above.

### Statistical analysis

The sample size was determined by a power analysis based on effect sizes from our previous experience with molecular and behavioral measures. Statistical testing was performed using Prism 8 (GraphPad, San Diego, CA, USA). Data were analyzed by one-way ANOVA, followed by post hoc Tukey’s or Kruskal–Wallis tests, or two-way ANOVA, followed by post hoc Sidak’s test. *p* < 0.05 was considered statistically significant. Statistical outliers were determined using the ROUT method and removed prior to performing ANOVAs. For correlations, two-tailed Pearson’s correlations were performed using PASW v.18 software (SPSS). Rat experiments (except for RNA-Seq) were repeated 2–3 times and the data are presented as the mean ± SEM from combined experiments.

## Results

### Increased expression of *Stat3* and its transcriptional target genes in the hippocampus of ethanol withdrawn rats after chronic exposure

**Fig. 1:**
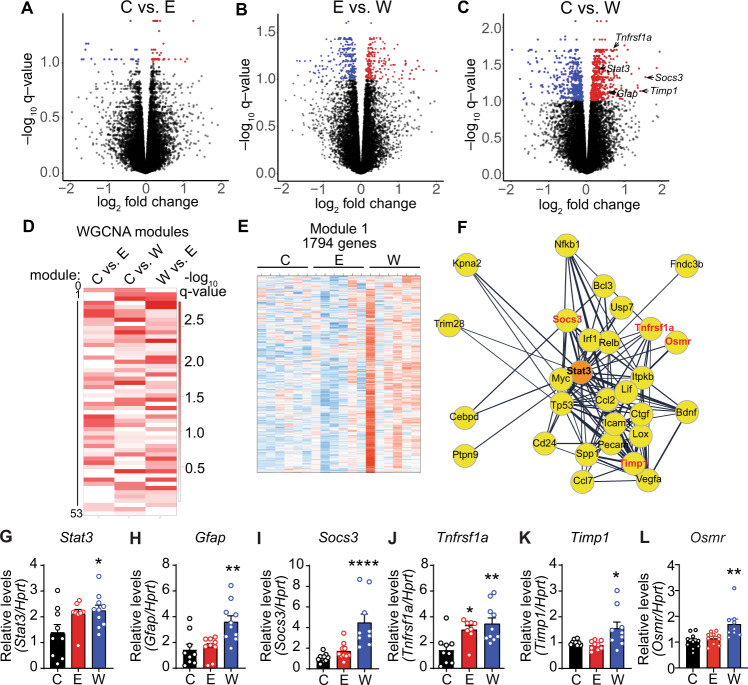
Increased expression of *Stat3* and its transcriptional target genes in the hippocampus of ethanol withdrawn rats after chronic exposure. Rats were fed control liquid diet (C), ethanol liquid diet (E), or ethanol liquid diet followed by 24 h of withdrawal from ethanol diet (W). The dorsal hippocampus was dissected and RNA isolated and subjected to RNA sequencing (RNA-Seq, *n* = 6) or qPCR (*n* = 10). **A**–**C** Volcano plots of genes differentially expressed between (**A**) C and E, (**B**) E and W, and (**C**) C and W by RNA-Seq. Red and blue dots indicate genes that were increased and decreased, respectively, in each comparison with *q* ≤ 0.1. **D** Heat map of 53 modules identified by weighted gene coexpression network analysis (WGCNA) with eigengene *q*-values (−log_10_) in C vs. E, C vs. W, and W vs. E comparisons. **E** Heat map showing expression of 1794 genes in module 1 in C, E, and W conditions. Each column is from an individual rat. Blue and red indicate decreased and increased expression levels, respectively. **F** Cytoscape plot of STRING protein–protein interaction analysis showing a module 1 cluster with the first shell of interactors with STAT3. **G**–**L** Relative expression by qPCR of (**G**) *Stat3*, (**H**) *Gfap*, (**I**) *Socs3*, (**J**) *Tnfrsf1a*, (**K**) *Timp1* and (**L**) *Osmr* in C, E, and W conditions. Data are presented as the mean ± SEM. *p < 0.05, **p < 0.01, and ****p < 0.0001 by Tukey’s test after one-way ANOVA.

We performed RNA-Seq to discover gene expression changes in the hippocampus of rats in withdrawal from chronic alcohol exposure. RNA-Seq was performed on the hippocampus from control, ethanol (no withdrawal), and ethanol plus withdrawal (24 h) groups. The largest number of differentially expressed (DE) genes were in the control vs. withdrawal comparison (925 out of 18,118 detected; *q* < 0.1), while only 65 genes were DE between control and chronic ethanol, and 474 genes were DE between chronic ethanol and withdrawal (Fig. 1A–C). These results indicate that more genes are altered in the hippocampus during withdrawal from chronic ethanol than during chronic ethanol exposure.

We next performed WGCNA^39^ to identify modules of co-expressed genes, with the rationale that members of a module could be regulated by a common molecular mechanism. We identified 53 modules (Fig. S1 and Table S1), with many of these modules exhibiting a significant association with at least one of the treatment conditions (Fig. 1D). The module 1 (1794 genes) eigengene value was significantly higher during withdrawal when compared with the control and ethanol conditions (Fig. 1E and Fig. S2, *p* < 0.05), and 186 genes in module 1 were elevated during withdrawal with a *q* value cutoff of 0.1 when compared with the control condition (Table S2). The top 5 enriched KEGG pathways for these 186 genes were TNF signaling, spliceosome, Kaposi’s sarcoma-associated herpesvirus infection, PI3K-Akt signaling, and human papillomavirus infection (Table S3), indicating alterations in the immune response and RNA splicing. We next performed a STRING analysis^37^ on these 186 genes to determine if there were any known protein–protein interactions and observed two clusters of highly interconnected genes. Genes in one cluster encoded RNA splicing factors, consistent with a top enriched pathway by KEGG. The second cluster contained the transcription factor STAT3 as a central node that was highly interconnected with other genes in the cluster (Fig. 1F). Many of these genes are known transcriptional targets of STAT3, including *Socs3*, *Tnfrsf1a*, *Timp1*, *Icam*, *Ctgf*, and *Osmr*^40–45^. Closer examination of the 186 DE genes in module 1 also revealed increased expression of *Gfap* during withdrawal. GFAP is a marker for reactive astrocytes and another transcriptional target of STAT3^46^. These results indicate altered RNA splicing, immune response, and STAT3 signaling in the hippocampus during withdrawal from chronic alcohol exposure.

To validate the RNA-Seq results, we performed qPCR of *Stat3*, *Gfap*, *Tnfrsf1a*, *Timp1*, and *Osmr* in hippocampal RNA from the three treatment groups and found that the expression of these genes was significantly elevated in the withdrawal compared to the control group (Fig. 1G–L). *Tnfrsfa1* expression was also higher in the chronic ethanol compared to the control group (Fig. 1J). Thus, the expression of *Stat3* and some of its known transcriptional target genes are elevated in the hippocampus during ethanol withdrawal after chronic exposure.

### Increased total and phosphorylated STAT3 protein in the hippocampus of ethanol withdrawn rats after chronic exposure

**Fig. 2:**
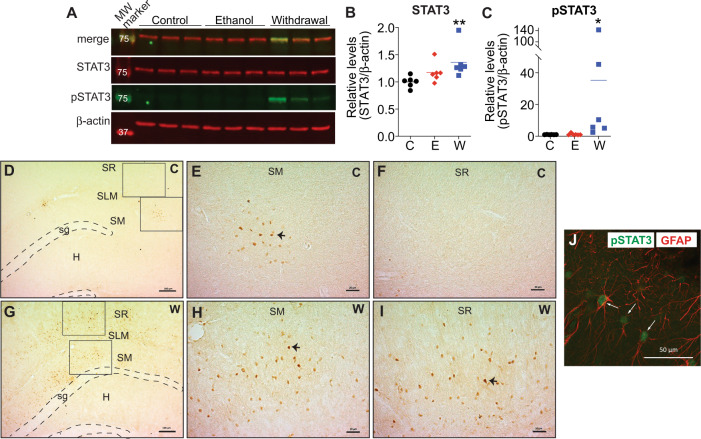
Increased total and phosphorylated STAT3 protein in the hippocampus of ethanol withdrawn rats after chronic exposure. Rats were fed control liquid diet (C), ethanol liquid diet (E), or ethanol liquid diet followed by 24 h of withdrawal from ethanol diet (W). The dorsal hippocampus was either (**A**–**C**) dissected and protein lysates analyzed by western blot (*n* = 6 per group) or (**D**–**J**) processed for immunohistochemistry (*n* = 3 per group). **A** Representative western blot showing (top to bottom) merged image of phosphorylated (p)STAT3 and STAT3, STAT3 (red), pSTAT3 (green), and β-actin (red). Molecular weight markers are indicated in kDa. **B** Levels of STAT3 relative to β-actin. **C** Levels of pSTAT3 relative to β-actin. Data are presented as the mean ± SEM. *p < 0.05 and **p < 0.01 when compared with control by Kruskal–Wallis test after one-way ANOVA. Representative images of hippocampus showing pSTAT3-positive cells in brown in (**D**–**F**) control and (**G**–**I**) ethanol withdrawn rats. Panels (**D**) and (**G**) are images at ×5 magnification with a scale bar representing 100 μm. Boxes show areas that are magnified in (**E**–**F**) and (**H–I**), with scale bar representing 50 μm. sg stratum granulosum, SM stratum moleculare, SLM stratum lacunosum moleculare, SR stratum radiatum, H hilus. **J** Representative immunofluorescent image of pSTAT3 in green, colocalized with the astrocytic marker glial fibrillary acidic protein (GFAP) in red. White arrows show localization of pSTAT3 in a GFAP-expressing cell. Scale bar, 50 μm.

To determine if increased *Stat3* mRNA during ethanol withdrawal is functionally relevant, we measured protein levels of STAT3 and pSTAT3^42^ in the hippocampus of control, ethanol, and ethanol-withdrawn rats by western blot. Total STAT3 and pSTAT3 were significantly higher in the ethanol withdrawal compared to the control group (Fig. 2A–C). We examined the hippocampal structures that contained pSTAT3 using IHC and observed pSTAT3 in the stratum moleculare and stratum radiatum (Fig. 2D–I and Fig. S3), areas that are enriched in astrocytes^47^. It is interesting to note that pSTAT3 was not strictly limited to the hippocampus and was observed in other regions such as the hypothalamus in the same sections (data not shown). We next examined whether pSTAT3 was located in astrocytes, microglia, or neurons in the hippocampus by performing immunofluorescent staining with antibodies to pSTAT3 and either the astrocytic maker, GFAP, the microglial marker, IBA1, or the neuronal marker, NeuN. Nuclear pSTAT3 was observed in cells immunoreactive for GFAP (Fig. 2J), but not NeuN (Fig. S4). pSTAT3 was also observed in IBA1-positive cells, although only 9% of pSTAT3-positive cells were also labeled with IBA1 (Fig. S4), compared with 86% of pSTAT3-positive cells labeled with GFAP. These results indicate that elevations in pSTAT3 in the hippocampus during withdrawal from chronic alcohol may be abundant in astrocytes.

### Increased pSTAT3 enrichment at target genes in the hippocampus of ethanol withdrawn rats after chronic exposure

**Fig. 3:**
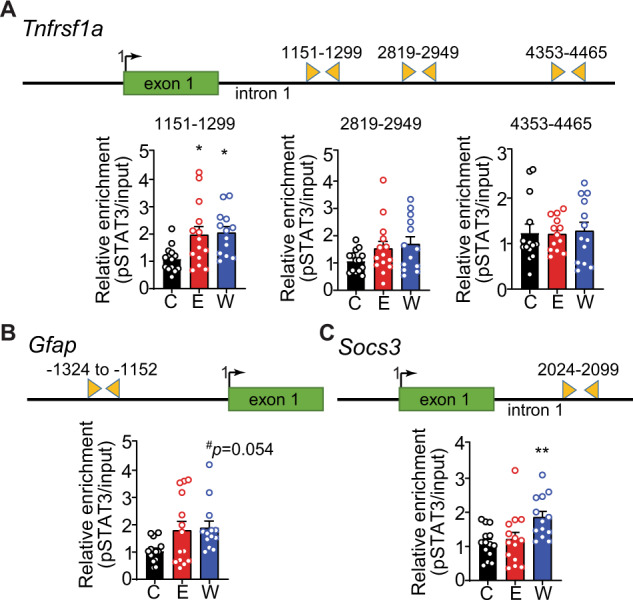
Increased pSTAT3 enrichment at target genes in the hippocampus of ethanol withdrawn rats after chronic exposure. Rats were fed control liquid diet (C, *n* = 13), ethanol liquid diet (E, *n* = 14), or ethanol liquid diet (*n* = 13) followed by 24 h of withdrawal from ethanol diet (W). The dorsal hippocampus was dissected and chromatin immunoprecipitation (ChIP) was performed using an antibody to phosphorylated STAT3 (pSTAT3), followed by qPCR with primers surrounding predicted STAT3 binding sites. Shown above each graph is a schematic of each gene with primer locations indicated by triangles. **A** Bar diagram shows relative pSTAT3 enrichment in the first intron of *Tnfrsf1a* between 1151 and 1299 bp downstream of the transcription start site but not at sites further downstream (2819–2949 and 4353–4465). Bar diagrams show relative pSTAT3 enrichment at the **B** *Gfap* promoter and **C** first intron of *Socs3*. Data are presented as the mean ± SEM. *p < 0.05 and **p < 0.01 compared with control by Tukey’s test after one-way ANOVA.

We hypothesized that the increase in pSTAT3 in the hippocampus of rats in ethanol withdrawal would result in enrichment of pSTAT3 at those genes whose expression was increased during withdrawal. To test this, we performed ChIP with an antibody to pSTAT3, followed by qPCR with primers flanking known or predicted STAT3 consensus sequences in *Tnfrsf1a*, *Gfap*, and *Socs3*. pSTAT3 was significantly enriched at a predicted STAT3 motif in the first intron of *Tnfrsf1a*, 1192 bp downstream of the transcriptional start site, in both the ethanol and withdrawal groups (Fig. 3A), consistent with the elevated mRNA levels of *Tnfrsf1a* under both conditions (Fig. 1J). Enrichment of pSTAT3 at this location was specific, as pSTAT3 was not enriched at two other predicted STAT3 binding motifs located further downstream. pSTAT3 was also more highly enriched during withdrawal at a STAT3 motif in the *Gfap* promoter located 1214 bp upstream of the transcriptional start site (Fig. 3B), and at a STAT3 motif located in the first intron of the *Socs3* gene (Fig. 3C). These results demonstrate that pSTAT3 associates with specific target genes during alcohol withdrawal and implicates pSTAT3 in transcriptional activation of these genes during withdrawal.

### Treatment with a STAT3 inhibitor during withdrawal decreases anhedonia

**Fig. 4:**
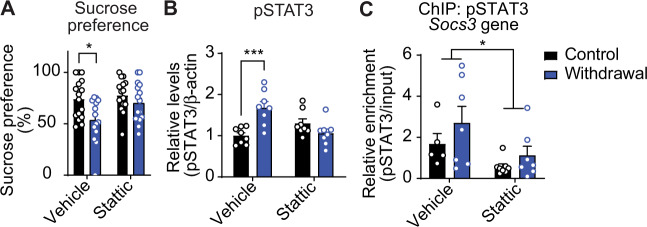
Treatment with a STAT3 inhibitor during withdrawal decreases anhedonia as measured by the sucrose preference test. Rats were fed control or ethanol liquid diet for 15 days followed by withdrawal for 24 h. Stattic (50; mg/kg, i.p.) or vehicle was administered 2 h prior to testing anhedonia. Hippocampus was dissected immediately after behavioral testing and analyzed for phosphorylated STAT3 (pSTAT3) by western blot and enrichment of pSTAT3 at the *Socs3* promoter using chromatin immunoprecipitation (ChIP)-qPCR. **A** Sucrose preference test, *n* = 14–16. *p < 0.05 by Sidak’s test after two-way ANOVA (stattic, *F*_(1, 58)_ = 4.46, *p* = 0.039; withdrawal, *F*_(1, 58)_ = 8.88, *p* = 0.0042; interaction, *F*_(1, 58)_ = 1.88, *p* = 0.18). **B** pSTAT3 protein levels, *n* = 8. ***p < 0.001 by Sidak’s test after two-way ANOVA (stattic × withdrawal interaction, *F*_(1, 28)_ = 17.5, *p* = 0.0003). **C** Enrichment of pSTAT3 at *Socs3* gene after ChIP-PCR, *n* = 5–8. *p < 0.05, main effect of stattic by two-way ANOVA (*F*_(1, 23)_ = 6.56, *p* = 0.018). Data are presented as the mean ± SEM.

To determine if blocking STAT3 activity reduces anhedonia during withdrawal from chronic alcohol, we treated rats with the STAT3 inhibitor stattic (50 mg/kg, i.p), 22 h into ethanol withdrawal, and tested them for anhedonia using the sucrose preference test 2 h later. Vehicle-treated rats in ethanol withdrawal had decreased sucrose preference compared to vehicle-treated control rats. Treatment with stattic normalized sucrose preference in rats in ethanol withdrawal (Fig. 4A), indicating that STAT3 inhibition decreased anhedonia during withdrawal. After the completion of the sucrose preference test, we verified the efficacy of stattic by measuring levels of pSTAT3 and enrichment of pSTAT3 at the *Socs3* gene by western blot and ChIP, respectively. As expected, pSTAT3 in the hippocampus of vehicle-treated rats in ethanol withdrawal was higher than in vehicle-treated, control diet-fed rats, while pSTAT3 in the hippocampus of rats treated with stattic during ethanol withdrawal was comparable to control-diet fed rats (Fig. 4B). pSTAT3 was enriched at the *Socs3* gene in vehicle-treated rats and decreased by stattic treatment (Fig. 4C). These results indicate that inhibition of STAT3 can reduce ethanol withdrawal-induced anhedonia.

### Increased phosphorylated (p)STAT3 and expression of STAT3 target genes in the postmortem hippocampus of human subjects with AUD

**Fig. 5:**
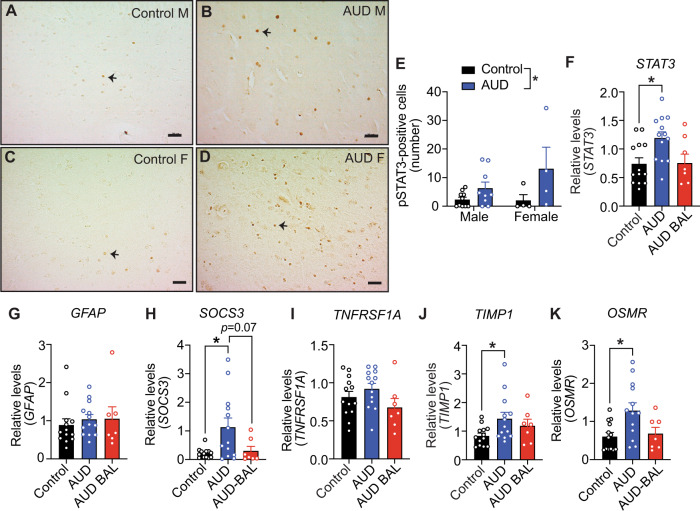
Increased phosphorylated (p)STAT3 and expression of STAT3 target genes in the postmortem hippocampus of human subjects with AUD. **A**–**D** Representative hippocampal sections showing immunohistochemistry with a pSTAT3 antibody (brown spots) from (**A**, **C**) control and (**B**, **D**) AUD subjects. Arrows point to examples of nuclei positive for pSTAT3. Scale bar, 50 μm. M male, F female. **E** Quantification of pSTAT3-positive cells in control and AUD subjects. Data are presented as the mean ± SEM, *n* = 10 males per group and 4 females per group. *p < 0.05 main effect of condition by two-way ANOVA (*F*_(1, 23)_ = 6.7, *p* = 0.016). **F**–**K** Relative expression of **F** *STAT3*, **G** *GFAP*, **H** *SOCS3*, **I** *TNFRSF1A*, **J** *TIMP1*, and **K** *OSMR* by qPCR in human postmortem hippocampus of control (*n* = 13), AUD (*n* = 13), and AUD subjects with blood alcohol levels at the time of death (AUD-BAL, *n* = 7). *p < 0.05 when compared to control by post hoc Tukey’s test after one-way ANOVA.

We next investigated whether our findings in rats translate to humans by performing IHC with pSTAT3 antibody on postmortem hippocampus from 14 controls and 14 AUD subjects. AUD subjects had significantly lower body mass index, higher ethanol daily use at the time of death, drinks per week, and cigarette pack-years compared with controls (Table 1). Notably, the number of pSTAT3-positive nuclei in the hippocampus was significantly higher in individuals with AUD (Fig. 5). We reasoned that an increase in activated STAT3 would reflect elevated expression of its target genes in the hippocampus of AUD subjects. To test this, we compared DE genes from previously published microarray data of hippocampus from human AUD and control subjects^48^ (GSE44456) with the DE genes in our rat withdrawal vs. control comparison. Gene homologs were matched based on gene symbols, and concordant gene pairs were determined based on an FDR < 0.2 and log_2_ fold changes in the same direction for both species. We found 39 concordant DE genes between the rat and human data (Fig. S5, Table S4). Several of these genes were present in rat WGCNA module 1, including *TNFRSF1A*, *TAGLN2*, *PECAM1*, *OSMR*, *IFITM2*, and *SLC14A1*. Specifically, the STAT3 target genes, *TNFRSF1A*^43^ and *OSMR*^45^, were significantly DE in both rat and human hippocampus. Other STAT3 targets, *SOCS3*^40^ and *TIMP1*^41^, were also concordantly DE in the rat and human datasets, although they did not meet the FDR < 0.2 cutoff in our analysis (*q* = 0.23 for both).

We extended these studies in human postmortem hippocampus from AUD and control subjects by performing qPCR to measure expression of *STAT3*, *GFAP*, *SOCS3*, *TNFRSF1A*, *TIMP1*, and *OSMR* mRNA in human AUD (*n* = 20) and control (*n* = 13) hippocampus (Table 1, Fig. 5). The AUD samples were divided based on whether there were measurable blood alcohol levels (BALs) at the time of death (13 AUD and 7 AUD-BAL [mean BAL = 0.221 ± 0.062%; range = 0.03–0.43%]) because of differences in gene expression that we observed in the rat hippocampus in the ethanol and withdrawal conditions. *STAT3*, *SOCS3*, *TIMP1*, and *OSMR* levels were significantly increased in AUD compared to control hippocampus, but not in AUD-BAL compared to control hippocampus (Fig. 5). Next, we examined correlations between *STAT3* and *GFAP*, *SOCS3*, *TNFRSF1A*, *TIMP1*, and *OSMR* transcript levels in all samples. *STAT3* transcript was significantly correlated with *TNRFSF1A* (*r* = 0.777, *p* < 0.01), *SOCS3* (*r* = 0.746, *p* < 0.01), *OSMR* (*r* = 0.922, *p* < 0.01), and *TIMP1* (*r* = 0.772, *p* = 0.01) transcripts (Fig. S6), suggesting coregulation of these genes. The correlations remained significant in the control, AUD, and AUD-BAL groups even when the groups were analyzed separately (Table S5). STAT3 expression was not significantly correlated with alcohol drinking measures of total drinking years, ethanol daily use, or a number of drinks per week (Table S5). Overall, our results provide evidence for activation of the STAT3 signaling pathway in the hippocampus of individuals with AUD similar to our preclinical findings, and suggest that this signaling pathway could be a target for AUD treatment.

## Discussion

Using a genome-wide transcriptomic approach, we identified STAT3 as a key transcriptional regulator in the rat hippocampus that is involved in anhedonia during withdrawal from chronic alcohol drinking. Activation of STAT3 during alcohol withdrawal in the hippocampus primarily occurred in astrocytes, as demonstrated by the majority of pSTAT3-positive cells colocalizing with the reactive astrocyte marker GFAP. Of note, the results we obtained using a preclinical model of dependence translate to humans, as an increased number of pSTAT3-labeled cells was observed in the hippocampus of human subjects with AUD compared with control subjects and known STAT3 target genes (*SOCS3*, *TIMP1*, and *OSMR*) were expressed at higher levels in the hippocampus of AUD subjects. These results indicate that STAT3 signaling is activated not only in the rat hippocampus after chronic ethanol exposure, but also in the hippocampus of individuals with AUD.

The RNA-Seq data pointed to the enrichment of genes in several biological pathways in the rat hippocampus during alcohol withdrawal, including those involved in RNA splicing and the response to viral infection (i.e., immune response pathways). These results are consistent with transcriptomic profiling of the hippocampus of mice in withdrawal from chronic intermittent ethanol vapor exposure^49,50^. Notably, the transcriptome of postmortem hippocampus of human subjects with AUD has been analyzed and, similar to our results, changes in genes related to RNA processing and immune response pathways have been found^48,51^. The behavioral implications and molecular details of altered expression of RNA splicing factors have not been investigated in the context of chronic alcohol exposure and withdrawal, although Wolfe et al.^52^ demonstrated that the synaptic transcriptome is changed in the hippocampus of mice treated acutely with ethanol, resulting in differential exon usage. Chronic alcohol exposure and withdrawal, by changing the expression of splicing factors, could conceivably alter exon usage, leading to alterations in synaptic proteins and ultimately impacting depression-like behavior. This requires further investigation.

With regard to the enrichment of immune response pathways in our rat RNA-Seq data, activation of the neuroimmune system after chronic alcohol exposure is a common theme across species and has been observed in rodent models of AUD and in postmortem brain from humans with AUD^16,17,48,53^. Here, we determined that STAT3 protein and mRNA levels increase, that STAT3 is activated, and that *Tnfrsf1a* (encoding tumor necrosis factor receptor 1A), *Osmr* (encoding oncostatin M receptor), and *Socs3* (encoding suppressor of cytokine signaling 3) are STAT3-regulated immune-response genes that are induced in the rat hippocampus upon chronic alcohol exposure and withdrawal. A previous study of the human hippocampal AUD transcriptome reported increased expression of the STAT3 target genes *TNFRSF1A*, *SOCS3, TIMP1*, and *OSMR*^48^ in AUD subjects vs. controls, consistent with our findings showing increased expression of most of these genes in the hippocampus of AUD vs. control subjects. Given that we observed increased pSTAT3 in the hippocampus of AUD subjects, it seems likely that the increased expression of these genes in the human AUD hippocampus is mediated by activated STAT3. Thus, STAT3 is a transcriptional mediator of neuroimmune activation in the hippocampus in response to chronic alcohol exposure. Of note, the increase in pSTAT3 was highly variable during withdrawal in rat hippocampus and in the hippocampus of human subjects with AUD. The source of this variability is not known but could reflect innate differences in the neuroimmune response to chronic alcohol use and withdrawal.

The majority of pSTAT3 in the hippocampus co-localized with GFAP, demonstrating that STAT3 activation primarily occurs in astrocytes in response to withdrawal from chronic alcohol. Further evidence for astrocytic activation of STAT3 during alcohol withdrawal is provided by our finding that pSTAT3 is enriched at the *Gfap* promoter. GFAP is a marker of reactive astrocytes and a known STAT3 target gene^46^. Astrocytes are key mediators of the brain immune response^54^, and STAT3 is considered an important regulator of astrocyte reactivity^55^. Reactive astrocytes exhibit transcriptional states that have been defined as neurotoxic, called “A1”, or neuroprotective, called “A2”^56,57^. A1 astrocytes are found in many neurodegenerative and autoimmune diseases, whereas A2 astrocytes have been observed after ischemic stroke^57^. STAT3 and GFAP have been described as “pan-injury” markers of reactive astrocytes^57,58^. STAT3 can play a protective or detrimental role after nervous system injury or neurodegeneration^59–61^. It remains to be determined whether the increase in pSTAT3 in hippocampal astrocytes after chronic alcohol drinking and withdrawal is pro- or anti-inflammatory, and whether it might contribute to alcohol-induced neurodegeneration and related behavioral sequelae.

It is important to note that although we observed increased pSTAT3 in astrocytes, there was also a small percentage (<10%) of pSTAT3 observed in microglia in the rat hippocampus during withdrawal from chronic alcohol. We cannot rule out the possibility that microglial-expressed STAT3 may be involved in the neuroimmune^62^ and anhedonic responses during withdrawal from chronic alcohol. Depression has been postulated to be a disease mediated by microglia^63,64^. Indeed, knockout of the *Stat3* gene in microglia alleviates depression-like behavior in mice^27^. Further investigation will be necessary to disentangle whether activation of STAT3 signaling in microglia, astrocytes, or both cell types, promotes depression-like behavior during withdrawal from chronic alcohol exposure.

*Socs3* was one STAT3 target gene whose expression increased during withdrawal from chronic alcohol exposure. *Socs3* transcription increases after cytokine receptor engagement and STAT3 activation. SOCS3 acts in a negative feedback loop to inhibit STAT3 and provide a molecular brake on an overactive immune response^65,66^. The induction of *Socs3* during withdrawal from chronic alcohol drinking might be a mechanism of limiting pro-inflammatory signaling. Consistent with its role as a negative regulator of STAT3, knockout of *Socs3* in mouse astrocytes have the opposite effect of *Stat3* knockout on inflammation in a spinal cord injury model^67^. Further investigation is necessary to understand the role of SOCS3 in alcohol withdrawal-induced neuroinflammation and the fine balance between STAT3 and SOCS3 signaling in pro- vs. anti-inflammatory signaling in the brain in response to alcohol. Interestingly, expression of *Lif* and *Osmr*, both encoding STAT3 activators^68,69^, were increased during alcohol withdrawal and in WGCNA module 1 (Fig. 1F and Table S2). They could be involved in activating STAT3 in the hippocampus during alcohol withdrawal.

The behavioral relevance of activated STAT3 in the hippocampus during alcohol withdrawal was established using STAT3 inhibitor (stattic) treatment. Rats in alcohol withdrawal exhibited an anhedonic phenotype, as reported by us earlier^10^. Interestingly, treatment with stattic alleviated alcohol withdrawal-induced anhedonia, indicating that activated STAT3 may promote anhedonia. Targeting STAT3 pharmacologically has the potential to dampen an overactive neuroimmune response in AUD and treat comorbid AUD and depression in humans. Although stattic is not suitable for use in humans, other STAT3 inhibitors are being developed for cancer therapy and other disease indications^70^ and could conceivably be repurposed for AUD.

## Supplementary information

Supplementary Methods and Figures

Supplementary Table S1

Supplementary Table S2

Supplementary Table S3

Supplementary Table S4

Supplementary Table S5
