# Supplementary material for: Transcriptomics identifies STAT3 as a key regulator of hippocampal gene expression and anhedonia during withdrawal from chronic alcohol exposure
Source: Transl Psychiatry. 2021 May 20;11:298. doi: 10.1038/s41398-021-01421-8 (PMC8170676; doi:10.1038/s41398-021-01421-8)
Supplement: Supplementary file 1 — Supplementary Methods and Figures [file 41398_2021_1421_MOESM1_ESM.pdf]

Supplementary Methods and Results to accompany:

Transcriptomics identifies STAT3 as a key regulator of hippocampal gene expression and anhedonia during withdrawal from chronic alcohol exposure

Wei-Yang Chen, PhD[1], Hu Chen, PhD[1], Kana Hamada, PhD[1], Eleonora Gatta, PhD[1], Ying Chen, MD[1], Huaibo Zhang, MD, PhD[1], Jenny Drnevich, PhD[2], Harish R. Krishnan, PhD[1], Mark Maienschein-Cline, PhD[1,3], Dennis R. Grayson, PhD[1], Subhash C. Pandey, PhD[1,4], and Amy W. Lasek, PhD[1*]

[1]Center for Alcohol Research in Epigenetics, Department of Psychiatry, University of Illinois at Chicago, Chicago, IL 60612 USA

[2]Roy J. Carver Biotechnology Center, University of Illinois at Urbana-Champaign, Urbana, IL 61801 USA

[3]Research Informatics Core, University of Illinois at Chicago, Chicago, IL 60612 USA

[4]Jesse Brown VA Medical Center, Chicago, IL 60612 USA

*Correspondence: Amy W. Lasek, Center for Alcohol Research in Epigenetics, Department of Psychiatry, University of Illinois at Chicago, 1601 W Taylor ST, MC 912, Chicago, IL 60612. Email: alasek@uic.edu; Tel: (312) 355-1593.

Contents

1. Supplemental Materials and Methods
2. Supplemental Figures
   a. Figure S1: WGCNA dendrogram
   b. Figure S2: WGCNA module 1 eigengene plot
   c. Figure S3: Quantification of pSTAT3-expressing cells in rat hippocampus.
   d. Figure S4: Fluorescence immunohistochemistry of pSTAT3/NeuN and pSTAT3/IBA1
   e. Figure S5: Scatterplot of DE genes in rat ethanol withdrawal vs control and human AUD vs. controls hippocampus.
   f. Figure S6: Correlations between expression of *STAT3* and *TNFRSF1A*, *SOCS3*, *TIMP1*, and *OSMR*.
3. References

1. Supplemental Materials and Methods

RNA-Seq and bioinformatics

Total RNA was isolated using the miRNeasy Mini Kit (Qiagen) and RNA integrity numbers were determined using a TapeStation instrument (Agilent, Santa Clara, CA, USA). RNA integrity numbers were all above 8, indicating high-quality RNA. cDNA libraries were prepared for sequencing using TruSeq RNA Library Preparation Kit (Illumina, San Diego, CA, USA) and were paired end sequenced on the HiSeq 4000 System (Illumina). The libraries were prepared and sequenced in the DNA Services laboratory of the Roy J. Carver Biotechnology Center at the University of Illinois at Urbana-Champaign. Approximately 100,000,000 raw reads were obtained per sample and were aligned to the NCBI Rnor 6.0 genome (annotation 106) using STAR (v. 2.5.2a). Approximately 98% of reads aligned to the genome, out of which 63% were mapped to a gene using featureCounts (v. 1.5.0). A total of 18,118 genes had edgeR's[1] TMM-normalized values > 0.5 and were kept for differential expression analysis. After adjusting for surrogate variables[2], we used limma-voom[3] to calculate a one-way ANOVA across all 3 treatments and obtained the raw *p* value and false discovery rate (*q*). Weighted gene co-expression network analysis (WGCNA) was performed in order to group genes into modules showing similar expression pattern across samples. Principle components analysis of the genes in the modules was used to calculate the "eigengene" value. Clusters or modules with similar expression patterns were merged, resulting in a total of 53 modules. STRING[4] was used to identify physical (direct) and functional (indirect) protein-protein association networks. Enrichr[5] was used for Kyoto Encyclopedia of Genes and Genomes (KEGG) pathway analysis.

Quantitative real-time PCR (qPCR)

For rat samples total RNA was converted to cDNA using iScript Reverse Transcription Supermix for RT-qPCR (Bio-Rad, Hercules, CA, USA). For human postmortem samples, total RNA was converted to cDNA using M-MLV Reverse Transcriptase (Invitrogen). qPCR was performed using SsoAdvanced Universal SYBR Green Supermix (Bio-Rad). Relative mRNA levels were determined using the $2^{-\Delta\Delta Ct}$ method with 2 reference genes (*Hprt* and *Gusb*) for rat samples and 3 reference genes for human samples (β2-microglobulin [*B2M*], glyceraldehyde-3-phosphate dehydrogenase [*GAPDH*], and β-actin [*ACTB*]). Rat data are shown normalized to *Hprt*, as relative mRNA levels did not differ when normalized to either *Hprt* or *Gusb* and human data are normalized to the 3 reference genes.

Rat primer sequences

Name	Experiment	Sequence (5'-3')
Tnfrsf1a-1151F	ChIP	CCCATTCCCTTCCTTTTCTC
Tnfrsf1a-1299R	ChIP	ATAGCCAGAAGGGGCAGTCT
Tnfrsf1a-2819F	ChIP	CCTTCCCTCTTTCCTTTGCT
Tnfrsf1a-2949R	ChIP	GGGTCTGGCTTCAAGAATCA
Tnfrsf1a-4353F	ChIP	CGCCCCATCTCTTTTGTTAC
Tnfrsf1a-4465R	ChIP	GCTAACCACTGGGTTCAAGG
Gfap-1324F	ChIP	CGGCAGAGAACAAGGCTCTA
Gfap-1152R	ChIP	CCATCTATCCCCTCCACTCA
Socs3-2024F	ChIP	GCCCCTCTTCCCACTCAG
Socs3-2099R	ChIP	TGAGTTGAACTGGGATTTGG
*Socs3*F	mRNA	CCCCGCTTTGACTGTGTACT
Socs3R	mRNA	AAAGGAAGGTTCCGTCGGTG
*Tnfrsf1a*F	mRNA	ACTTCATTCACCAGCGTTGC
*Tnfrsf1a*R	mRNA	GGGTGTATCCCCATCAGCAG
*Stat3*F	mRNA	TCGGAAAGTATTGTCGCCCC
*Stat3*R	mRNA	GACATCGGCAGGTCAATGGT
*Gfap*F	mRNA	GCGAAGAAAACCGCATCACC
GfapR	mRNA	GGTGGCCTTCTGACACAGAT
*Timp1*F	mRNA	CATCTCTGGCCTCTGGCATC

*Timp1*R	mRNA	GCCCTTATAACCAGGTCCGA
*Hprt*F	mRNA	TCCTCAGACCGCTTTTCCCGC
*Hprt*R	mRNA	TCATCATCACTAATCAGACGCTGG

Human primer sequences

Name	Experiment	Sequence (5'-3')
STAT3F	mRNA	CATGTCTCCTTGCGTGTCTAA
STAT3R	mRNA	CATGTCCAACCTGTAACTCTCTC
GFAPF	mRNA	GCCTCTGGATTGTGGGAATTA
GFAPR	mRNA	GGCCTTTAGAAATGGGACAAAG
SOCS3F	mRNA	CAAGACCTTCAGCTCCAAGAG
SOCS3R	mRNA	TCACTGCGCTCCAGTAGAA
TNFRSF1AF	mRNA	CTCCAAATGCCGAAAGGAAATG
TNFRSF1AR	mRNA	ATAATGCCGGTACTGGTTCTTC
TIMP1F	mRNA	GGATACTTCCACAGGTCCCAC
TIMP1R	mRNA	CAGGGGATGGATAAACAGGGA
OSMRF	mRNA	AGTTCCCTGAGCCAAATTTCT
OSMRR	mRNA	GCCTTCTTCCACCAGCTTAT
B2MF	mRNA	CTATCCAGCGTACTCCAAA
B2MR	mRNA	GCTCCACTTTTTCAATTCTC
ACTBF	mRNA	CTCCCTGGAGAAGAGCTAC
ACTBR	mRNA	GATCCACACGGAGTACTTG
GAPDHF	mRNA	CGAGATCCCTCCAAAATCAA
GAPDHR	mRNA	TTCACACCCATGACGAACAT

Immunohistochemistry (IHC)

After perfusion with 4% paraformaldehyde, brains were post-fixed overnight in 4% paraformaldehyde and cryoprotected in 30% sucrose for 2 days before freezing at -80°C. Coronal sections (40 µm) were cut on a cryostat and mounted onto glass slides. Slides were heated in SignalStain EDTA Unmasking Solution (Cell Signaling Technology, Danvers, MA, USA) for antigen retrieval. After blocking tissue in 5% normal donkey serum (Jackson ImmunoResearch,

West Grove, PA, USA) for 1 h at room temperature, slides were incubated with mouse monoclonal pSTAT3 antibody (1:200 dilution, #4113, Cell Signaling Technology; RRID: AB_2198588) overnight at 4°C, then incubated with biotinylated secondary antibody (#BA-9200, Vector Laboratories, Burlingame, CA, USA; RRID: AB_2336171) and developed using the VECTASTAIN Elite ABC-HRP kit (#PK-6100, Vector Laboratories, RRID: AB_2336819). For immunofluorescence, rat sections were incubated with pSTAT3 and GFAP, IBA1, or NEUN antibodies (pSTAT3, Cell Signaling Technology, RRID: AB_2198588; GFAP, Thermo Fisher Scientific #130300, RRID: AB_2532994; IBA1, Abcam #AB5076, RRID:AB_2224402; NEUN, Abcam #AB128886, RRID:AB_2744676) diluted in 5% normal donkey serum (Jackson ImmunoResearch), followed by AlexaFluor 488 or 594 labeled secondary antibodies (Jackson ImmunoResearch, #715-545-151, RRID: AB_2341099 and #712-585-153, RRID: AB_2340689). Brightfield images were acquired at 20x magnification using a Zeiss AxioScope A1 microscope (Carl Zeiss, NY, USA). Immunofluorescent images were captured at 60x magnification using a Zeiss LSM710 confocal microscope (Carl Zeiss). The number of pSTAT3-positive cells in the hippocampus were counted from 3 sections per rat and 3 rats per group or from 1 section per human subject using ImageJ software (National Institutes of Health).

Western blot

Frozen tissue was lysed and manually homogenized using a pestle in ice cold 1X RIPA buffer (Cell Signaling Technology) with 1X Halt protease and phosphatase inhibitors (Thermo Fisher Scientific), 1 mM sodium fluoride, and 1 mM sodium orthovanadate. Protein concentrations were determined using the BCA Protein Assay Kit (Thermo Fisher Scientific). Equal protein amounts (25 μg) were separated by gel electrophoresis on precast Novex 4-12%

Tris-glycine gels (Thermo Fisher Scientific) and transferred to nitrocellulose membranes using the Trans-Blot Turbo system (Bio-Rad). Membranes were blocked with Odyssey Blocking TBS Buffer (LI-COR, Lincoln, NE, USA) and incubated with primary (4°C, overnight) and secondary (1 h, room temperature) antibodies in 5% BSA in TBST (20 mM Tris-HCl, 150 mM NaCl, pH 7.4, 0.1% Tween-20). Primary antibodies were the following: STAT3, Cell Signaling Technology #9139, RRID: AB_331757, 1:1000 dilution; phosphorylated STAT3 (pSTAT3), Cell Signaling Technology #9145, RRID: AB_2491009; and β-actin, Sigma-Aldrich #A5441, RRID: AB_476744, 1:10,000 dilution. Secondary antibodies were IRDye 680RD donkey anti-mouse IgG, LI-COR Biosciences #925-68072, RRID: AB_2814912, 1:10,000 dilution; and IRDye 800CW donkey anti-rabbit IgG, LI-COR Biosciences #925-32213, RRID: AB_2715510, 1:5000 dilution. Blots were imaged on the Odyssey Fc Dual-Mode Imaging system (LI-COR) and analyzed using Image Studio Lite (LI-COR).

2. Supplemental Figures

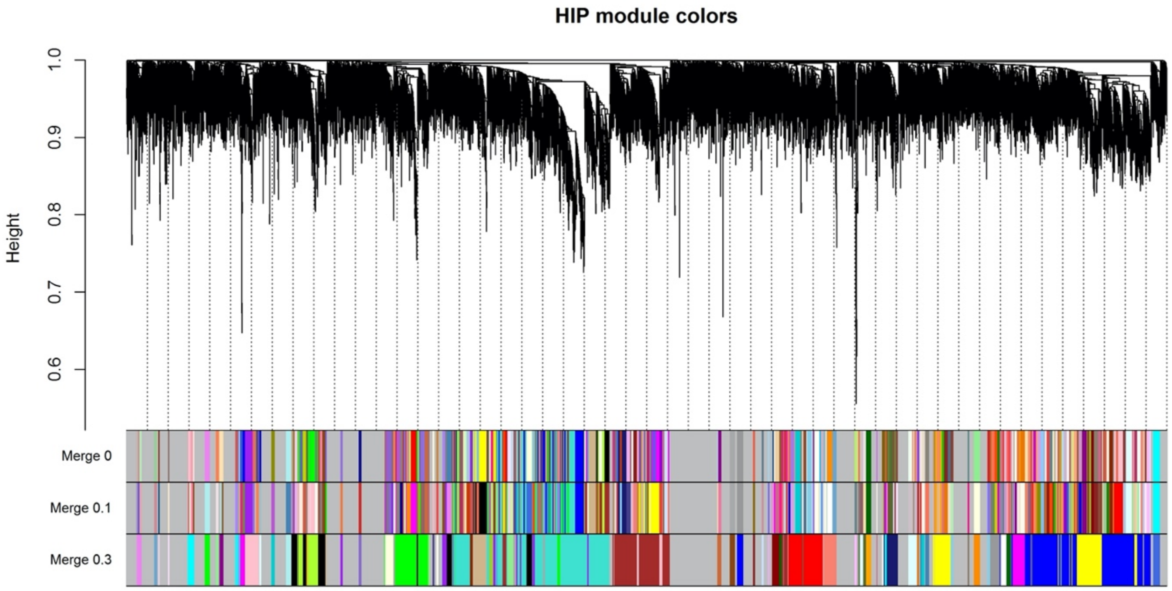

Fig. S1. Dendrogram for WGCNA modules showing merge at 0.3. Module colors are shown at the bottom.

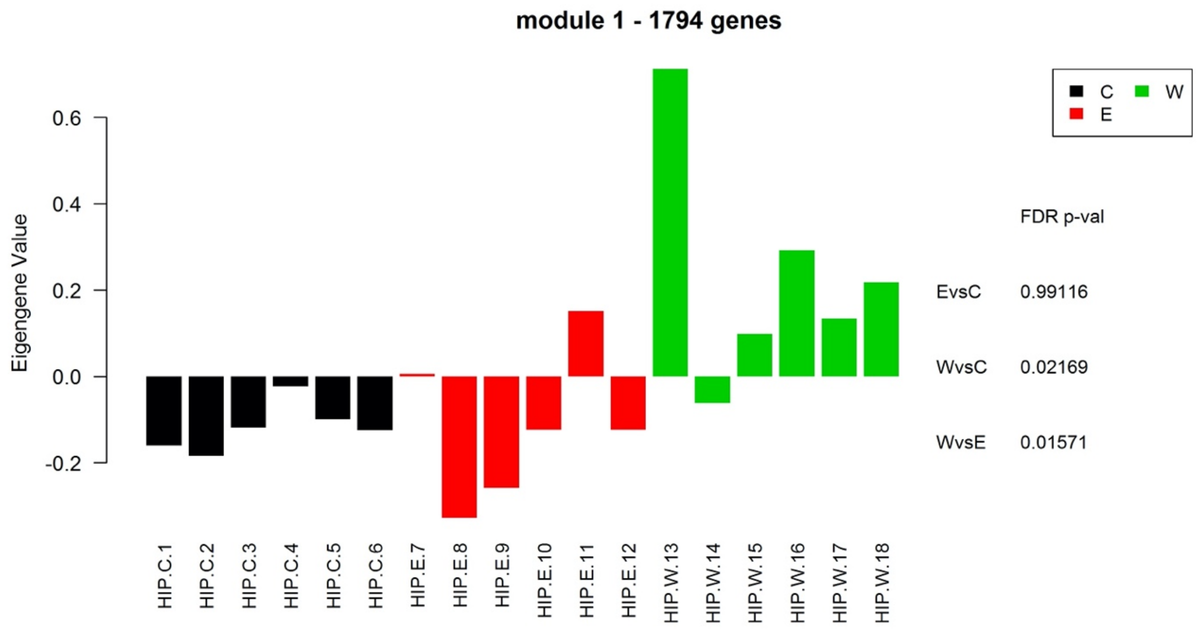

Fig. S2. Eigengene values for WGCNA module 1 for each individual rat in control (C, black), ethanol (E, red), and withdrawal (W, green) conditions, n=6. The False Discovery rate (FDR)-adjusted *p*-value for each comparison is shown on the right. W vs. C and W vs. E comparisons were significantly different.

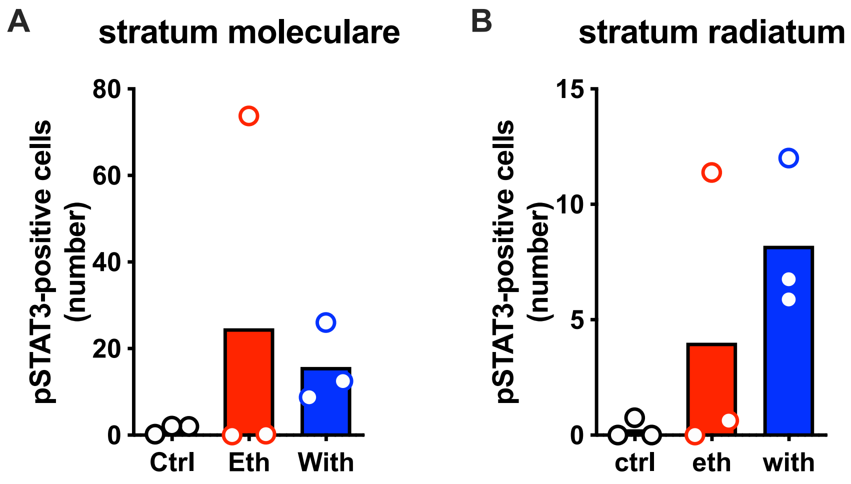

Fig. S3. Quantification of pSTAT3 in rat hippocampus sections. The number of pSTAT3-positive nuclei were counted in the (**a**) stratum moleculare and (**b**) stratum radiatum from 3 sections per rat and 3 rats per group. Each point is the average of 3 sections per rat.

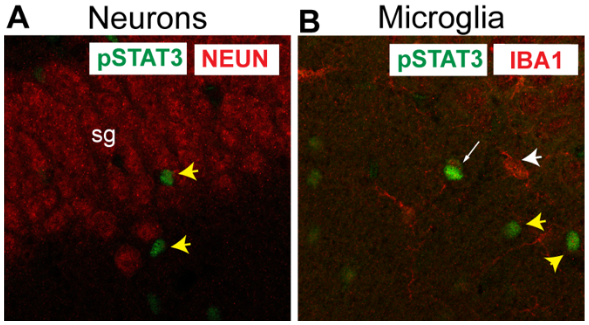

Fig. S4. Immunofluorescence of rat hippocampal sections showing (A) pSTAT3 in green and NeuN in red, and (B) pSTAT3 in green and IBA1 in red. sg, stratum granulosum. Yellow arrowheads indicate cells that are only labelled with antibody to pSTAT3. White arrow in panel (B) shows a cell that is labelled with pSTAT3 and IBA1 antibodies and white arrowhead shows an IBA1-labelled cell that is not pSTAT3 positive.

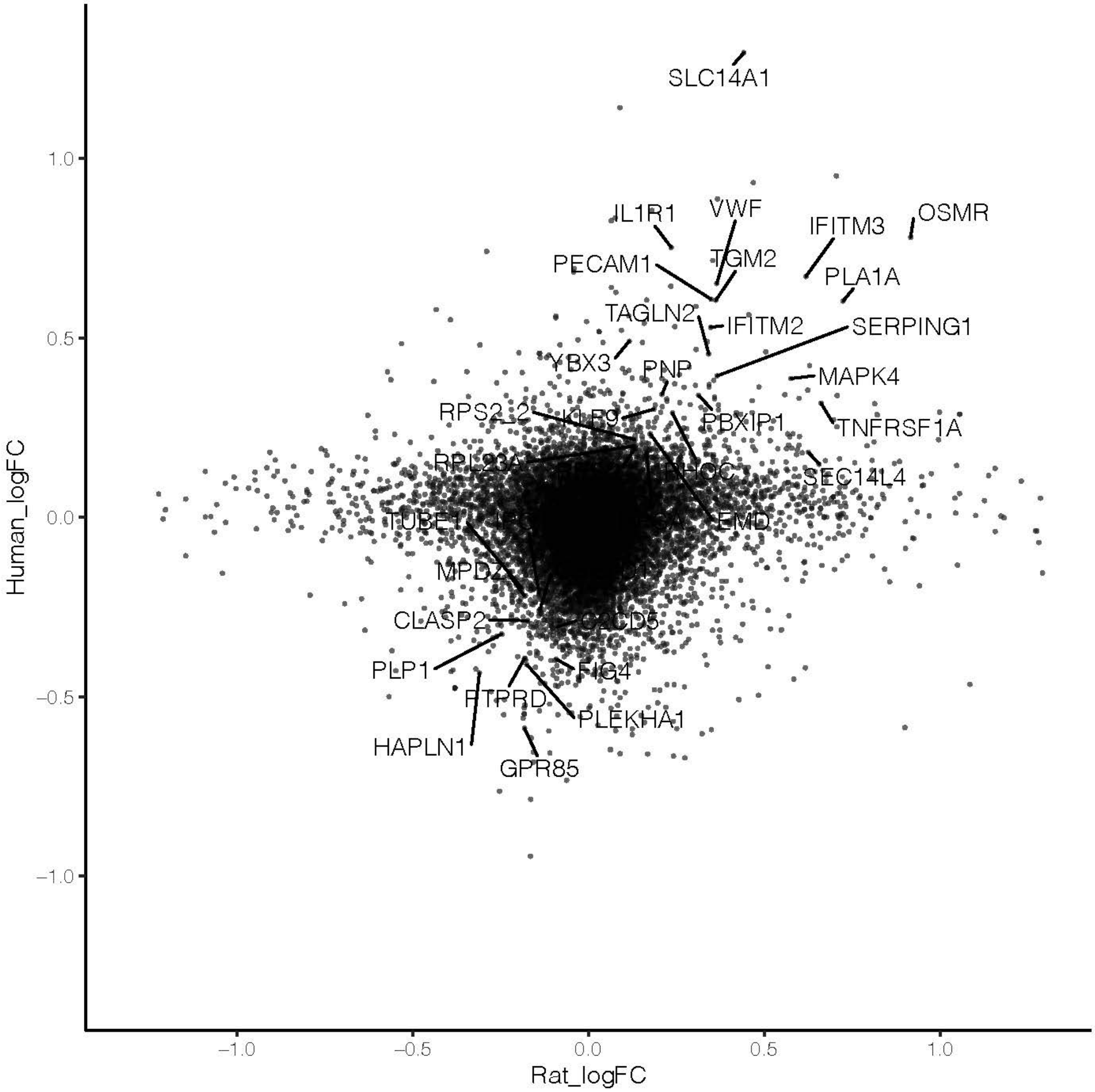

Fig. S5. Scatterplot of differentially expressed genes (log fold change) in rat ethanol withdrawal vs. control (x-axis) and human AUD vs. control (y-axis) hippocampus.

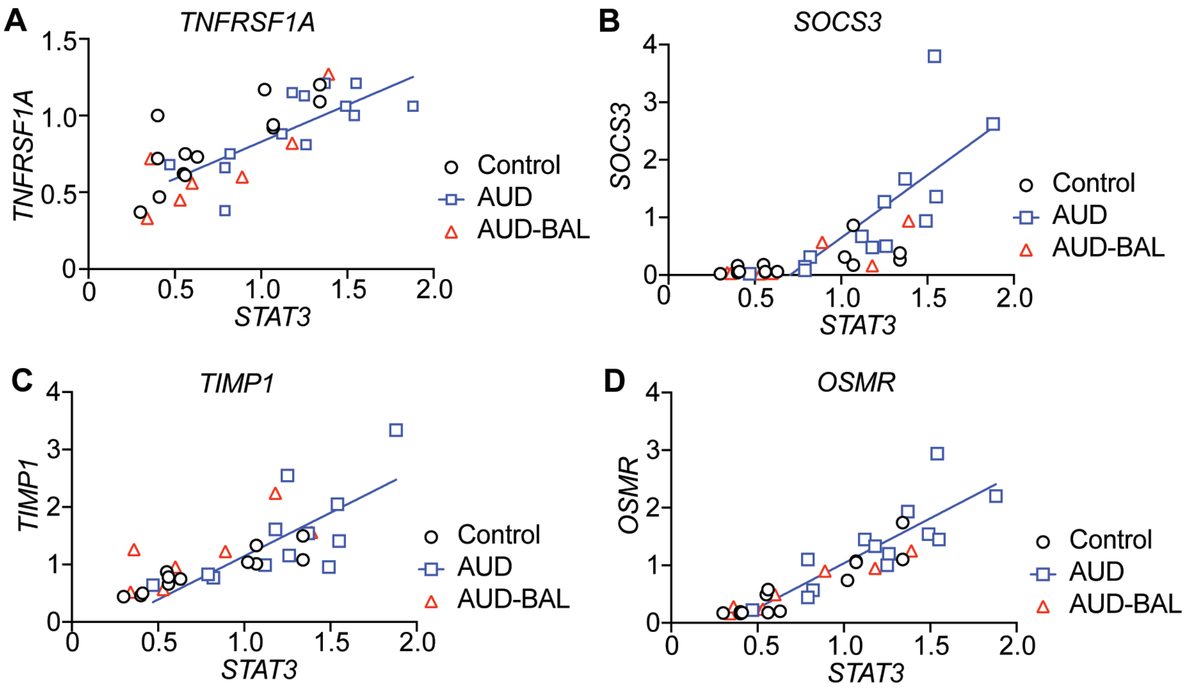

Fig. S6. Correlations between expression of *STAT3* and **a** *TNFRSF1A*, **b** *SOCS3*, **c** *TIMP1*, and **d** *OSMR*. AUD samples were divided into groups based on whether there were measureable blood alcohol levels at the time of death (AUD-BAL).

References

1. Chen Y, Lun AT, Smyth GK. From reads to genes to pathways: differential expression analysis of RNA-Seq experiments using Rsubread and the edgeR quasi-likelihood pipeline [version 2; peer review: 5 approved]. *F1000Res* 2016; **5:** 1438.

2. Leek JT, Storey JD. Capturing heterogeneity in gene expression studies by surrogate variable analysis. *PLoS Genet* 2007; **3**(9)**:** 1724-1735.

3. Law CW, Chen Y, Shi W, Smyth GK. voom: Precision weights unlock linear model analysis tools for RNA-seq read counts. *Genome Biol* 2014; **15**(2)**:** R29.

4. Szklarczyk D, Gable AL, Lyon D, Junge A, Wyder S, Huerta-Cepas J *et al.* STRING v11: protein-protein association networks with increased coverage, supporting functional discovery in genome-wide experimental datasets. *Nucleic Acids Res* 2019; **47**(D1)**:** D607-D613.

5. Kuleshov MV, Jones MR, Rouillard AD, Fernandez NF, Duan Q, Wang Z *et al.* Enrichr: a comprehensive gene set enrichment analysis web server 2016 update. *Nucleic Acids Res* 2016; **44**(W1)**:** W90-97.
